# Distal regulation of *c-myb* expression during IL-6-induced differentiation in murine myeloid progenitor M1 cells

**DOI:** 10.1038/cddis.2016.267

**Published:** 2016-09-08

**Authors:** Junfang Zhang, Bingshe Han, Xiaoxia Li, Juraj Bies, Penglei Jiang, Richard P Koller, Linda Wolff

**Affiliations:** 1 Key Laboratory of Aquacultural Resources and Utilization, Ministry of Education, College of Fishery and Life Science, Shanghai Ocean University, No.999 Huchenghuan Road, Pudong New District, Shanghai 201306, China; 2 Laboratory of Cellular Oncology, Center for Cancer Research, National Cancer Institute, Bethesda, MD 20892, USA

## Abstract

The c-Myb transcription factor is a major regulator that controls differentiation and proliferation of hematopoietic progenitor cells, which is frequently deregulated in hematological diseases, such as lymphoma and leukemia. Understanding of the mechanisms regulating the transcription of *c-myb* gene is challenging as it lacks a typical promoter and multiple factors are involved. Our previous studies identified some distal regulatory elements in the upstream regions of *c-myb* gene in murine myeloid progenitor M1 cells, but the detailed mechanisms still remain unclear. In the present study, we found that a cell differentiation-related DNase1 hypersensitive site is located at a −28k region upstream of *c-myb* gene and that transcription factors Hoxa9, Meis1 and PU.1 bind to the −28k region. Circular chromosome conformation capture (4C) assay confirmed the interaction between the −28k region and the *c-myb* promoter, which is supported by the enrichment of CTCF and Cohesin. Our analysis also points to a critical role for Hoxa9 and PU.1 in distal regulation of *c-myb* expression in murine myeloid cells and cell differentiation. Overexpression of Hoxa9 disrupted the IL-6-induced differentiation of M1 cells and upregulated *c-myb* expression through binding of the −28k region. Taken together, our results provide an evidence for critical role of the −28k region in distal regulatory mechanism for *c-myb* gene expression during differentiation of myeloid progenitor M1 cells.

c-Myb is a transcription factor that regulates hematopoiesis by controlling essential cellular processes, including proliferation, survival, and differentiation.^1, 2^ Dysregulated expression of *c-myb* has been implicated in the pathogenesis of leukemia, as in human T-cell leukemia (T-ALL) *c-myb* was found to be involved in translocation and duplication.^3, 4^ Dysregulation of *c-myb* also has roles in human colon and breast carcinoma.^5, 6, 7^ Additional studies demonstrate that overexpression or mutations in *c-myb* can release its oncogenic potential, especially in myeloid cells.^8, 9^

*c-myb* transcription is regulated through several mechanisms. *c-myb* mRNA elongation can be blocked in intron I in a cell-type-dependent manner.^10, 11, 12^ c-Myb can negatively regulate its own expression.^13^ WT1 (Wilms Tumor 1), MZF1 (myeloid zinc finger 1), and PU.1 can downregulate the *c-myb* promoter activity, while the Ets and c-Jun/JunD transcription factors activate the promoter.^14, 15, 16, 17, 18, 19^ But these data from previous studies cannot completely elucidate the mechanisms of *c-myb* expression regulation during the differentiation of murine myeloid leukemia M1 cell.

Recent studies indicate that the expression of *c-myb* gene is also under control of distal regulatory elements, which is critical for its regulatory function. It has been reported that a transgene integration approximately 77 kb upstream of *c-myb* gene decreased the expression of *c-myb* gene in megakaryocyte/erythrocyte lineage-restricted progenitors, probably by disrupting an enhancer there, resulting in hematopoietic abnormalities including anemia, thrombocythemia, and splenomegaly.^20^ It has also been reported recently that distal enhancers located at −36 kb/−81 kb upstream of *c-myb* regulate gene expression by a long-range interaction with gene promoter during mouse erythroid development.^21^ Meanwhile, some integration sites of retroviruses in leukemia and lymphomas cluster have been located to upstream regions within 25–250 kb of *c-myb* gene, and integration of retroviruses at these sites is closely related to increased expression of *c-myb* gene and induction of neoplasia.^22, 23, 24, 25^ In murine myeloid progenitor M1 cells (M1 cells), we previously identified three murine leukemia virus integration regions, named Mml1 (murine leukemia virus-induced myeloid leukemia 1) (−25k), Mml2 (−56k), and Mml3 (−70k).^24^ These sites are proximal to the 5′ regulatory region of *c-myb* gene through DNA looping and facilitate the integrated virus to activate *c-myb* gene expression from far away.^26^ The above data suggest essential roles of distal elements in the regulation of *c-myb* gene expression, but detailed mechanisms involved in this process largely remain to be elucidated.

In our previous work,^26^ enrichment of active histone modifications H3K4me3 and H3K4me1 (hallmarks of enhancers) was found in the Mml1, Mml2 and Mml3 regions upstream of *c-myb* gene. These regions are proximal to the 5′ regulatory region of *c-myb* gene through DNA looping, indicating that these regulatory elements are involved in *c-myb* expression regulation. In the present study, we show that the regulatory element at the −28 kb region has an essential role in *c-myb* gene regulation during interleukin-6 (IL-6)-induced differentiation in M1 cells, and transcription factors (Hoxa9 and PU.1) and structural proteins (CCCTC-binding factor (CTCF), Cohesin) are involved in this process. We also demonstrate potential genome-wide distal interaction regions using circular chromosome conformation capture (4C) technology. The present work provides more details about the long-range regulatory mechanism of *c-myb* during differentiation of M1 cells.

## Results

### DNase I hypersensitive site analysis of the *c-myb* locus during IL-6-induced differentiation of M1 cells

**Figure 1:**
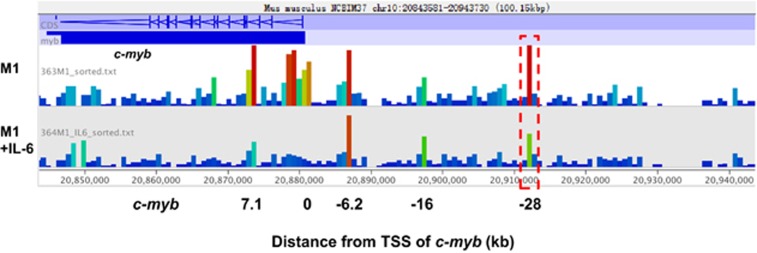
DNase I hypersensitive (DHS) site analysis of the *c-myb* locus in M1 and differentiated M1 cells. Nuclei from both M1 cells and IL-6-treated M1 cells were subjected to a limited DNase I digestion. Fractions containing small DNA fragments representing two-hit DNase I cutting events were precipitated, pooled, and prepared for paired-end sequencing. DHS-seq data of the *c-myb* locus are shown by the reads density created by the Seqmonk software in M1 and differentiated M1 cells. Red dashed box shows detected DHS site located at the −28 region during IL-6-induced differentiation. TSS, transcription start site

Our previous data indicated the existence of regulatory elements in the *c-myb* upstream region in M1 cells, based on the enrichment of active histone modifications H3K4me1 and H3K4me3.^26^ Here sequencing of DNase I hypersensitive sites (DHS-seq) was used to identify the open sites of the upstream region of *c-myb* gene in M1 cells. As shown in Figure 1, DNase I hypersensitive sites were detected in the *c-myb* promoter and distal upstream regions (−6.2k, −16k, −28k), with the highest signal peak at the gene-coding region. Open sites at +7.1k and the promoter region show the active *c-myb* gene transcription.

It has been suggested that quantitative comparison of DNase I hypersensitivity sites between states is highly useful in predicting transcription factor occupancy associated with particular biological processes. As *c-myb* expression goes down during differentiation of M1 cells induced by IL-6 treatment, DHS-seq was also performed using IL-6-treated M1 cells. The *c-myb* expression decreased after IL-6 treatment in a time-dependent manner (Supplementary Figure S1). It is interesting that DNase I hypersensitivity sites at Mml1 region (−28k) reduced remarkably after IL-6 treatment (Figure 1), suggesting the existence of active/functional regulatory elements in the Mml1 region that are involved in regulation of *c-myb* gene during IL-6-induced differentiation in M1 cells. To our surprise, we did not detect open sites at −56k and −70k (data not shown), which are also virus insertion sites named Mml2 and Mml3. In our previous study, interactions of −56k and −70k regions with the *c-myb* promoter did not change when *c-myb* was downregulated.^26^ Therefore, we focused on the Mml1 region for further study.

### Transcriptional factors bind to *c-myb* upstream Mml1 region in a cell differentiation-dependent manner

**Figure 2:**
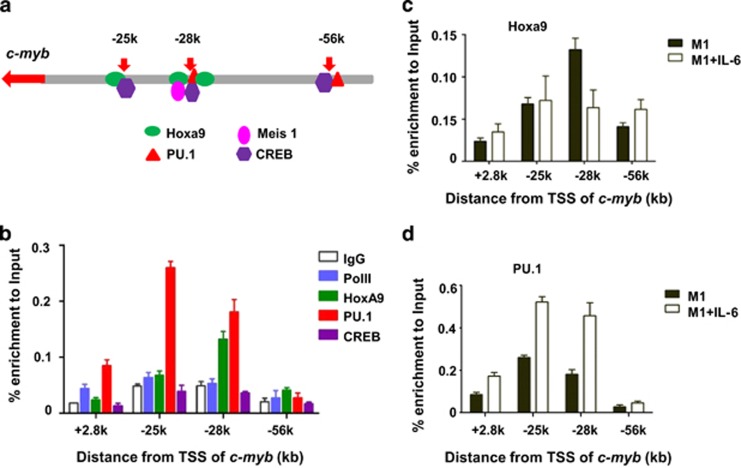
Binding of transcription factors to upstream regions of the *c-myb* locus. (**a**) Potential transcription factor-binding sites of the *c-myb* locus are predicted; (**b**) ChIP-qPCR was performed to investigate the binding of the indicated transcription factors to the indicated regions of the *c-myb* locus in M1 cells; (**c**) the binding of Hoxa9 and (**d**) PU.1 to the indicated regions during IL-6-induced differentiation in M1 cells was also detected using ChIP-qPCR. Results are representative of three independent experiments. TSS, transcription start site

The regulation of gene expression requires binding of regulatory proteins to cis-regulatory elements. Computational method, TFSEARCH, was used for predicting potential transcription factor binding to the Mml1 region. Hoxa9, Meis1, PU.1, and CREB were predicted to bind at the target regions as shown in Figure 2a. The roles of those proteins in regulation of *c-myb* gene were investigated using chromatin immunoprecipitation (ChIP).

M1 cells were prepared for ChIP using antibodies against Pol II, Hoxa9, PU.1, and CREB, with PCR primer sets for sites in +2.8k, −25k, −28k, and −56k regions, according to the enrichment of H3K4m1 (Supplementary Figure S2) and predicted binding sites in those regions. As shown in Figure 2b, Pol II is substantially enriched at +2.8k as expected, Hoxa9 is enriched at the sites in−28k and −56k regions with substantial enrichment at −28k, and PU.1 is substantially enriched at the sites in +2.8k, −25k, and −28k regions. Enrichment of CREB is not observed in this study.

To further assess the involvement of the above proteins in regulation of *c-myb* expression during differentiation of M1 cells, ChIP was performed in M1 cells treated or not treated with IL-6. As shown in Figure 2c, after a 5-day IL-6 treatment the enrichment of Hoxa9 significantly reduced in the −28k region, but not in other regions, suggesting that the −28k region is a major region for Hoxa9 regulation of *c-myb* expression in IL-6-induced differentiation in M1 cells. The enrichment of PU.1 significantly increased in the +2.8k, −25k, and −28k regions (Figure 2d). Our data indicate that transcription-relevant proteins bind to the distal upstream regions of the *c-myb* locus, and the binding may be involved in regulation of *c-myb* expression during IL-6-induced differentiation in M1 cells.

### Scanning of distal regulatory elements interacting with the *c-myb* promoter

**Figure 3:**
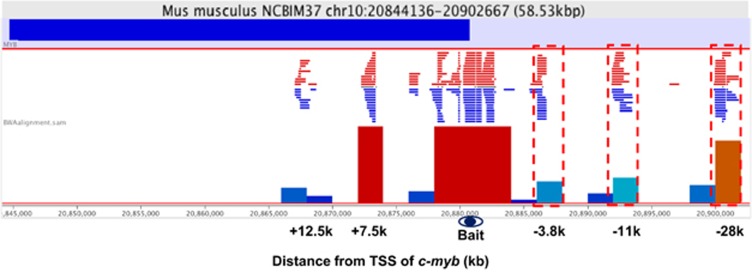
Long-range interaction between the *c-myb* promoter and distal regions. 4C assay was performed in M1 cells. A Csp6I fragment of 1364 bps containing the *c-myb* promoter was used as bait fragment. The bait region was mapped on the read count distribution plot generated by the Seqmonk software to visualize proximity of the peaks to the bait. The cross-linking frequency between the upstream regions and the bait fragment was presented by reads density. Interaction upstream regions of *c-myb* are marked by red dashed boxes. Distance of marked interaction regions from transcription start site (TSS) is shown as indicated

Our previous data from quantitative chromosome conformation capture assay (3C-qPCR) indicated long-range interactions between distal regions within 100 kb upstream of *c-myb* gene and the 5′ end of *c-myb* gene through DNA looping.^26^ To further understand the long-range DNA contacts involved in the *c-myb* locus, 4C assay, a technology to investigate genome-wide DNA contacts with a given genomic site of interest, was applied. The *c-myb* promoter fragment served as a bait fragment. As shown in Figure 3, three prominent peaks of interactions were detected around −3.8k, −11k, and −28k upstream of *c-myb* gene, which is consistent with our previous 3C data ^26^, indicating that 4C data are reliable and 4C method is suitable to detect long-range interactions of the *c-myb* promoter. The highest peak is located at the −28k region, indicating the interaction of this region with the *c-myb* promoter. We further scanned the potential regulatory elements interacting with the *c-myb* promoter at the genome-wide level. Besides the *c-myb* locus, 18 loci showed a high interaction frequency with the *c-myb* promoter with a reads count >500 (Supplementary Table S1). Ten of them are unknown intergenic regions. It is interesting to investigate these unknown interaction regions in our future work.

### Cohesin and CTCF modulate interaction between distal regulatory elements and the *c-myb* promoter

**Figure 4:**
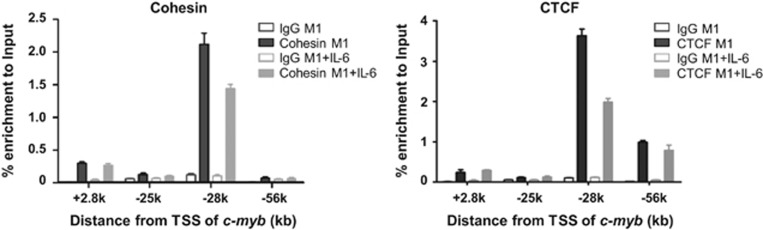
Enrichment of Cohesin and CTCF at upstream regions of the *c-myb* locus. ChIP using antibodies specific for Cohesin or CTCF was performed in M1 and differentiated M1 cells induced by IL-6. Primer sets specific for the regions of +2.8k, −25k, −28k, and −56k were used for quantitative PCR of ChIP DNAs. Relative quantitation was carried out by the comparative threshold cycle (CT) method. Statistical analysis was performed using the GraphPad Prism 5 software. Student's *t*-test was used on measurements of enrichment from three replicates. Error bars represent S.D. (*n*=3)

Cohesin and CTCF are central players in regulating long-range interactions,^27^ and our previous work showed that CTCF is recruited to the regions that interact with the *c-myb* promoter.^26^ Here we investigated the roles of Cohesin and CTCF in regulation of *c-myb* gene expression by IL-6 using ChIP-qPCR with antibodies against Rad21, a subunit of Cohesin, and CTCF. Cohesin is remarkably enriched at the −28k region (Figure 4a). CTCF is also strongly enriched at the −28k and −56k regions (Figure 4b). During IL-6 treatment, Cohesin and CTCF binding both decreased significantly at −28k and remained unchanged at other regions. The above data suggested that the binding of Cohesin and CTCF to −28k is involved in *c-myb* expression regulation during IL-6-induced differentiation in M1 cells.

### Hoxa9 upregulates *c-myb* transcription by binding to distal elements of the *c-myb* locus

**Figure 5:**
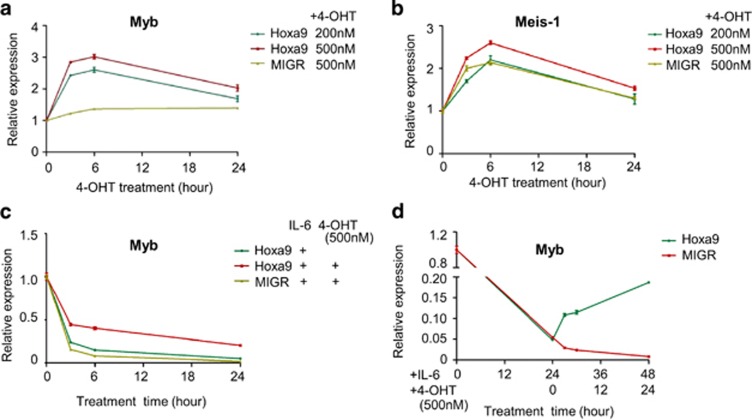
Hoxa9 upregulates *c-myb* in M1 cells. M1 cells were transfected with MIGR-Hoxa9-ER vector. Conditional activation of Hoxa9 was induced by addition of 4-OHT at the indicated doses in the presence of IL-6 or not. Then (**a**, **c**, **d**) *c-myb* and (**b**) *Meis-1* expression was detected using quantitative real-time PCR analysis. Data are normalized to GAPDH expression. MIGR: empty vector control. Error bars represent S.D. (*n*=3)

**Figure 6:**
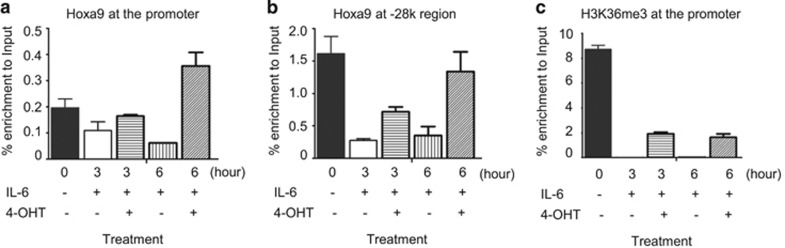
Hoxa9 binding at the distal element is associated with *c-myb* expression. M1 cells were transfected with MIGR-Hoxa9-ER vector and then Hoxa9 activation was induced by addition of 500 nM 4-OHT. The cells were treated with IL-6 or/and 4-OHT for the indicated times, then ChIP-qPCR was performed to investigate the enrichment of Hoxa9 at (**a**) the *c-myb* promoter and (**b**) the −28k region, and (**c**) the enrichment of H3K36me3 (active transcription marker) at the *c-myb* promoter. Relative quantitation was carried out by the comparative threshold cycle (CT) method. Statistical analysis was performed using the GraphPad Prism 5 software. Student's *t*-test was used on measurements of enrichment from three replicates. Error bars represent S.D. (*n*=3)

**Figure 7:**
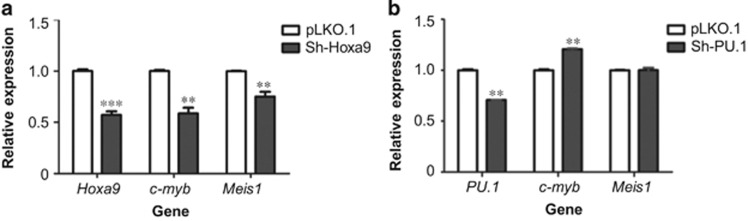
Regulation of *c-myb* expression by Hoxa9 and PU.1. M1 cells were infected with lentivirus containing (**a**) shRNA targeting Hoxa9 (sh-Hoxa9) or (**b**) PU.1 (sh-PU.1) or scrambled RNA (pLKO.1). Forty-eight hours later, mRNA levels of *c-myb* and *Meis1* were determined by quantitative reverse transcription PCR (RT-qPCR). Relative quantitation was carried out by the comparative threshold cycle (CT) method. Data are normalized to GAPDH expression. Statistical analysis was performed using the GraphPad Prism 5 software. Student's *t*-test was used on measurements of enrichment from three replicates. Error bars represent S.D. (*n*=3). An asterisk represents significant difference of expression (*P*<0.05). **P<0.01, ***P<0.001

It has been reported that c-Myb is an essential downstream target for homeobox-mediated transformation of hematopoietic cells.^28^ To further investigate the role of Hoxa9 in regulation of *c-myb* gene in M1 cells, the impact of Hoxa9 on *c-myb* expression in cytokine-treated M1 cells was analyzed. M1 cells were stably transfected with a MIGR1 vector expressing Hoxa9 fused to a modified estrogen receptor ligand-binding domain (Hoxa9-ER). Conditional activation of Hoxa9 was induced by addition of 4-hydroxytamoxifen (4-OHT). Supplementary Figure S3A shows the expression of Hoxa9-ER in M1 cells. Supplementary Figure S3B shows Hoxa9-ER mainly localized to the nuclear fraction after activation. Addition of 200 and 500 nM 4-OHT both increased *c-myb* (Figure 5a) and Meis-1 mRNA levels in M1 cells (Figure 5b). IL-6 treatment reduced *c-myb* expression in a time-dependent manner; however, overexpression of Hoxa9 counteracted the action of IL-6 (Figure 5c) and reversed IL-6-induced downregulation of *c-myb* expression (Figure 5d). ChIP-qPCR data show that Hoxa9 binding at the promoter and the −28k region decreased after IL-6 treatment (Figures 6a and b). Enrichment of Hoxa9 at −28k increased significantly after overexpression of Hoxa9 (Figure 6b) and was accompanied by increased expression of *c-myb*. Enrichment of H3K36me3 at the *c-myb* promoter supports the presence of positive transcriptional activity of the gene (Figure 6c). Meanwhile, lentivirus expressing shRNA targeting Hoxa9 or PU.1 was used to downregulate the expression of Hoxa9 or PU.1. Knockdown of Hoxa9 decreased the expression of *c-myb* and Meis1 (Figure 7a). On the contrary, knockdown of PU.1 increased *c-myb* expression and showed no significant effect on Meis1 transcription (Figure 7b). The above results suggest that Hoxa9 and PU.1 can regulate *c-myb* expression via binding of distal upstream elements.

## Discussion

c-Myb is a key regulator of hematopoiesis, abundantly expressed in the hematopoietic stem and progenitor cell compartments and downregulated as cells progress toward terminal differentiation. Although the function of c-Myb has been extensively studied, the mechanisms of its transcriptional regulation still remain unclear. Our previous data indicated the existence of distal regulatory elements in the *c-myb* upstream regions based on the enrichment of active histone modifications H3K4me3 and H3K4me1 and the demonstration of enhancer activity in upstream sequences from a region at −28k.^26^ Here we further investigate the mechanism of distal regulation of *c-myb* expression in differentiating M1 cells.

Genome-wide identification of regulatory elements based on chromatin accessibility and binding of regulatory factors were combined to investigate distal regulatory elements in this study. A DHS assay identified accessible sites in the distal upstream regions of the *c-myb* locus (Figure 1) and ChIP-qPCR assay showed that regulatory factors, Hoxa9, PU.1, and Meis1 are bound to these regions (Figure 2). Together with our previous work showing the enrichment of H3K4me1 and H3K4me3 at these sites and enhancer activity at −28k,^26^ our data strongly support the existence of regulatory elements in these upstream regions.

Evidence supporting long-range physical interactions between distal regulatory regions contained within 100 kb upstream of *c-myb* and the *c-myb* gene promoter region was provided in our previous study using 3C-qPCR data.^26^ The present 4C data not only confirmed this looping interaction between the upstream regions and the *c-myb* promoter but also identified additional genome-wide DNA sites as potential interaction partners of the *c-myb* locus (Supplementary Table S1). CTCF and Cohesin (Figure 4) are showed here to be involved in mediating the long-range interactions of distal regions of the *c-myb* locus. Interaction between distal regions within 100 kb and the *c-myb* promoter is closely related to *c-myb* gene expression, because most interactions appear only in *c-myb*-expressing M1 cells but not in NIH3T3 cells without *c-myb* expression.^26^ The present work also suggests that the distal regulatory regions are involved in *c-myb* expression regulation during M1 cell differentiation. DHS sites changed, long-range DNA interaction reduced, and the binding of regulatory factors at distal regions changed during IL-6-induced differentiation. Among these regions, the −28k region appears to be the most predominant site of regulation and the highest peak of Cohesin binding is also located at this site (Figure 4). In the study of mouse primary erythroid progenitors, long-range interactions were detected between −36 kb/−81 kb upstream of *c-myb* and the *c-myb* promoter, which regulate *c-myb* expression during erythroid development ^21^. However, we did not detect any interactions between −36 kb/−81 kb regions and the *c-myb* promoter in mouse myeloid progenitor M1 cells. It indicated that long-range interactions between distal enhancers and gene promoter are in a cell-specific manner.

The involvement of specific transcription factors in the regulation of *c-myb* through upstream regions was a focus in this study. As predicted from identification of sequence-binding sites, Hoxa9 was found to bind to −28k and −56k regions; however, binding at the −28k region reduced significantly during IL-6 treatment. The later indicates that Hoxa9 binding at the −28k region is closely related to regulation of *c-myb* gene expression during this cytokine-induced differentiation. Hoxa9 and Meis1 were reported to be positive regulators of *c-myb* in HPCs and leukemic cells,^28^ which was further confirmed here in M1 cells (Figure 5). Recently a genome-wide-level identification of binding sites for Hoxa9 and Meis1 in hematopoietic cells showed that Hoxa9 and Meis1 co-bind at hundreds of highly evolutionarily conserved sites, most of which are distant from transcription start sites, and many sites are also bound by PU.1 and other transcription factors.^29^

It has also been reported that Hoxa9 and Meis1 bind to the *c-myb* locus, mainly around the first intron region in HPC7 and FMH9 cells, and maintain its expression in HPCs and leukemic cells.^30^ However, although we observed moderate enrichment of Hoxa9 at +2.8k region, much stronger enrichment of Hoxa9 was located at −28k and −56k regions in M1 cells. Therefore, mechanisms to explain how *c-myb* gene expression is regulated by Hoxa9 in +2.8k region during cell differentiation remains to be explained. Our data also suggested that Hoxa9 is a positive regulator of Meis 1 expression, which is consistent with other researches.^31, 32^ And the detailed role of Hoxa9 in *c-myb* control during myeloid differentiation of human precursors remains unclear, although it has been reported that Hoxa9 can increase *c-myb* expression in human B-lineage-2 (BLIN-2) cell line.^33^

PU.1 has been reported to be a negative regulator of *c-myb* promoter activity,^17^ and our data confirm this in M1 cells (Figure 7). However, previous to our work here, its role in binding at distal regions of the *c-myb* locus was not documented. PU.1 binding was observed in this study at the +2.8, −25k, and −28k regions and increased during IL-6 treatment, suggesting the involvement of multiple sites for PU.1 in negative regulation of *c-myb* expression in differentiating M1 cells. But the detail mechanisms of PU.1 in *c-myb* control during human myeloid differentiation remain still unknown.

Increasing number of studies have suggested that distant transcriptional control has essential roles during cell differentiation. The roles of distal regulatory elements in gene regulation in the alpha and beta-globin loci have been well studied and reviewed.^34^ A distal DNA element within the imprinted mouse Igf2-H19 locus interacts with the Igf2 gene >100 kb away via chromatin looping or sliding during skeletal muscle differentiation.^35^ In the INK4b-ARF-INK4a locus, EZH2-dependent chromatin looping controls INK4a and INK4b but not ARF during human progenitor cell differentiation.^36^ A long-range interaction between the Il21 gene promoter and a distal enhancer (49 kb upstream) can be induced by IL-6 in CD4(+) T cells, resulting in induction of IL-21, STAT3, CTCF, and Cohesin to be involved in this process.^37^

The data presented here implicate important roles for transcriptional regulation in the intergenic regions upstream of *c-myb* during differentiation and leukemia. It is recently reported that distal regulatory elements at −71 kb and −84 kb upstream of *c-myb* gene and binding of some erythroid TFs, including LDB1, GATA 1, TAL1, ETO 2, and KLF1, have been reported to be involved in regulation of *c-myb* expression in human erythroid progenitors, suggesting the involvement of distal regulation in human *c-myb* expression.^38^ It will be of interest to determine whether these distal regions are altered by mutation or other mechanism involving looping in human leukemias.

## Materials and Methods

### Cell lines

The murine myeloid leukemia M1 cell (TIB 192; ATCC, Manassas, VA, USA) was maintained in RPMI 1640 medium with 10% heat-inactivated horse serum (Invitrogen, Carlsbad, CA, USA). For IL-6 treatment, M1 cells were seeded at a density of 1 × 10^5^ cells/ml in medium containing IL-6. IL-6 stocks were prepared as described previously.^26^ For conditional activation of Hoxa9, M1 cells were stably transfected with a MIGR-Hoxa9-ER vector expressing Hoxa9-ER,^29^ and Hoxa9 activation was induced by addition of 4-OHT (T176; Sigma-Aldrich, St. Louis, MO, USA).

### Quantitative real-time PCR analysis

Total RNA was isolated using TRIzol reagent (15596-018; Invitrogen). cDNA was produced from 1 μg of total RNA using a cDNA Reverse Transcription Kit (4322171; Applied Biosystems, Foster City, CA, USA). Quantitative real-time PCR was performed in triplicate with predesigned *c-myb* gene expression assays (Mm 00501741-m1; Applied Biosystems). Data were normalized to a mouse GAPDH (glyceraldehyde-3-phosphate dehydrogenase) control (4352932E; Applied Biosystems). Relative quantitation was carried out by the comparative threshold cycle (CT) method. Statistical analysis was performed using the GraphPad Prism 5 software (GraphPad software, San Diego, CA, USA). The Student's *t*-test was used on measurements of *c-myb* expression from three experimental replicates.

### ChIP assay

ChIP was performed as described before.^26^ In brief, cells were fixed in 0.8% formaldehyde for 6 min at room temperature. Sonicated chromatin was immunoprecipitated with antibodies for H3K4me1 (ab8895; Abcam, Cambridge, MA, USA), H3K36me3 (ab9050; Abcam), RNA polymerase II CTD (ab26721; Abcam), Rad21 (ab992; Abcam), PU.1 (sc-352X; Santa Cruz), CREB (ab31387; Abcam), Hoxa9 (sc-17155X; Santa Cruz Biotechnology, Santa Cruz, CA, USA), CTCF (ab70303; Abcam), or rabbit IgG (15006; Sigma-Aldrich). A 10% aliquot was removed as an input fraction. Quantitative real-time PCR was performed with ChIP DNA and input DNA using a Light Cycler 480II. Relative quantitation was carried out by the comparative threshold cycle (CT) method. Statistical analysis was performed using the GraphPad Prism 5 software. The Student's *t*-test was used on measurements of enrichment from different condition samples from three experimental replicates. Primers for ChIP-qPCR are shown in Table 1.

### Immunological methods and antibodies

For immunoblot, M1 cells were washed twice with cold phosphate-buffered saline (PBS), drained, and 0.5 ml of lysis buffer (20 mM Tris-HCl, pH 7.5, 150 mM NaCl, 1 mM Na_2_EDTA, 1 mM EGTA, 1% Triton X-100, 2.5 mM sodium pyrophosphate, 1 mM β-glycerophosphate, 1 mM Na_3_VO_4_ and 1 mg/ml leupeptin) supplemented with protease inhibitors was added to 5 × 10^6^ cells. The cells were then sonicated. After centrifugation at 4 °C and 15 000 × *g* for 10 min, protein aliquots containing 30 μg of protein were separated on denaturing and reducing Laemmli 12% polyacrylamide gels and transferred to nitrocellulose. The membrane was blocked in PBS containing 5% milk powder and 0.1% Tween 20 and incubated at 4 °C overnight with primary antibody and for 1 h at 25 °C with horseradish peroxidase-conjugated secondary antibody. Antibody binding was visualized using Clarity Western ECL Substrate (1705060; Bio-Rad, Hercules, CA, USA).

### Sequencing of DNase I hypersensitive sites

Intact nuclei were isolated from M1 and IL-6-treated M1 cells and digested with DNase I according to a standard protocol.^39^ Briefly, cells were harvested and washed twice in cold PBS and resuspended in Buffer A (15 mM Tris-HCl, pH 8.0, 15 mM NaCl, 60 mM KCl, 1 mM EDTA, 0.5 mM EGTA, 0.5 mM spermidine, and protease inhibitors (04693116001; Roche, Indianapolis, IN, USA)) at 2 × 10^6^ cells/ml. NP-40 (0.02%) was gently added and the suspension was incubated for 10 min on ice to release nuclei. Nuclei were washed twice in cold Buffer A and pelleted at 1200 × *g* for 10 min. Nuclei were treated with different concentrations (0, 40, 60, 80 U) of DNase1 (11284932001; Roche) in 2 ml of digestion buffer (15 mM Tris-HCl, pH 8.0, 15 mM NaCl, 60 mM KCl, 1 mM EDTA, 0.5 mM EGTA, 6 mM CaCl_2_) at 37 °C for 3 min. The reactions were stopped by the addition of 2 ml of stop buffer (50 mM Tris-HCl, pH 8.0, 100 mM NaCl, 0.1% SDS, 100 mM EDTA, 0.5 mM spermidine) containing 10 μg/ml RNase (11119915001, Roche). Samples were treated with 25 μg/ml proteinase K at 55 °C overnight. DNA was purified by gentle phenol/chloroform extraction and DNA fragments were separated by ultracentrifugation through a sucrose gradient. Fractions containing small DNA fragments representing two-hit DNase I cutting events were precipitated, pooled, and prepared for Single- Read sequencing according to the manufacturer Illumina (San Diego, CA, USA). Reads were trimmed to remove the adaptors and low-quality bases. High-quality sequences were aligned to the mouse genome (Mus musculus NCBIM37) by BWA alignment. At the genome-wide level, 167 313 DNase 1 hypersensitive sites were detected by reads count >21. DNase 1 hypersensitive sites at the *c-myb* locus were plotted by the Seqmonk software (Babraham Institute, Cambridge, UK). Raw data can be downloaded from the Gene Expression Omnibus (GEO) accession number GSE81031.

### 4C assay

4C assay was performed following a standard protocol.^40^ Briefly, cells were cross-linked by 1% formaldehyde for 10 min and 0.125 M glycine was added to prevent further cross-linking. Cells were collected and suspended in 100 μl of 0.125 M glycine–PBS per 10^6^ cells. One million cells were washed with 200 μl of digestion buffer and resuspended in 200ul of digestion buffer containing 0.3% SDS, incubated with shaking at 37 °C overnight. Next day, 400 μl of digestion buffer was added slowly. Samples were digested with 300 U Csp6I at 37 °C with shaking for 4 h and then an additional 300 U Csp6I was added and incubated at 37 °C with shaking for 4 h. In all, 200 U Csp6I was added and incubated at 37 °C with shaking overnight. After completing the assessment of digestion efficiency, SDS was added to a final concentration of 1.6%. Samples were incubated at 65 °C with shaking for 20 min to stop digestion and lyse the cells. Digested chromatin were collected and ligated in a very low DNA concentration (0.6 ng/μl) at 4 °C for 1 week with replenishing 60 μl of 100 mM ATP every third day. NaCl (final concentration 0.2 M), EDTA (final concentration 1 mM), and proteinase K (final concentration 0.2 mg/ml) were added to the ligation mix and reverse cross-linking was performed at 65 °C overnight. DNA was purified with the Qiagen PCR Purification Kit (28106; Qiagen, Hilden, Germany) as per the manufacturer's recommendation and treated with exonuclease I and exonuclease III. Circular DNA was then purified with the Qiagen PCR Purification Kit. A Csp6 I fragment of 1364 bps containing the *c-myb* promoter was used as a bait sequence. Two rounds of PCR were used for amplifying unknown potential interaction sequences. For the first round PCR, core PCRs were set up in triplicate for each circular DNA sample with core PCR primers (Forward primer: 5′-ACACTTCTGCCTTCAGGGTTT-3′, Reverseprimer: 5′-GAGGTGTGTTTGCACTTGAAGA-3′, 100-bp away from the Csp6I digestion site in the *c-myb* promoter bait sequence). For the second round PCR, core PCR products were diluted by 100-fold and PCRs in triplicate were set up for each diluted core PCR product with nested PCR primers (Forward primer: 5′-TCGGTTGAATAGAGTGAGCG-3′, Reverse primer: 5′-AGCCGTTTGAAGATTCTTGT-3′). The resulting nine nested PCR products were pooled and purified for paired-end sequencing according to the manufacturer Illumina. Reads started with one of the two 20-bp nested primer sequences selected and trimmed to remove the bait overhangs and low-quality bases. Finally, 96% of total reads of 59 095 377 was mapped to the mouse genome (Mus musculus NCBIM37) by BWA alignment. The bait region was mapped on the read count distribution plot to visualize proximity of peaks to the bait by Seqmonk tool. Distances from the transcription start site of *c-myb* gene to each of the peak regions upstream of *c-myb* gene were calculated. Raw data can be downloaded from the Gene Expression Omnibus (GEO) accession number GSE81031.

### Lentivirus production and cell transduction

DNA oligos for Hoxa9 (Forward: 5′-CCGGGCATTAAACCTGAACCGCTCTCTCGAGAGAGCGGTTCAGGTTTAATGCTTTTT-3′ Reverse: 5′-AATTAAAAAGCATTAAACCTGAACCGCTCTCTCGAGAGAGCGGTTCAGGTTTAATGC-3′) and for PU.1 (Forward: 5′-CCGGGCTTCCCTTATCAAACCTTGTCTCGAGACAAGGTTTGATAAGGGAAGCTTTTT-3′ Reverse: 5′-AATTAAAAAGCTTCCCTTATCAAACCTTGTCTCGAGACAAGGTTTGATAAGGGAAGC-3′) were synthesized by Shanghai Sangon Biotechnology (Shanghai, China). After annealing, the double-strand DNA was cloned into shRNA-expressing vector PLKO.1-puro. In all, 3 μg of appropriate packaging plasmids pCMV-VSVG: pCMV-DR 8.91 (1 : 5) and 5 μg of shRNA expressing vector were cotransfected into 2.5 × 10^6^ HEK-293T cells using PolyFect transfection reagent (301105; Qiagen). Seventy-two hours later, the media containing lentivirus particles were collected and centrifuged at 1500 × *g* for 10 min. Then the supernatant was collected and used to infect M1 cells immediately in the presence of 10 μg/ml Hexadimethrine Bromide (a.k.a. polybrene, H9268; Sigma-Aldrich). Forty-eight hours after infection, cells were collected for analysis.

### Data access

The data used in this study have been deposited in NCBI's Gene Expression Omnibus repository and are accessible through GEO accession number GSE81031.

## Figures and Tables

**Table 1:** Primer sets for ChIP-qPCR experiments

*Name*	*Forward primer (5′→3′)*	*Reverse primer (5′→3′)*
−25k	AGAAACTCAGAAGACCCTTTGC	GTCATGCCTGTTCTGTGTATGA
−28k	CACCTTTTGGTATGACATTTGAAC	AAAACAATGGAAAACCCCAG
−56k	TCTCTGACCCATTCCTCCTTTGCT	CCATGGTCCCTTGGTTCTGTTGTT
+2.8k	GAAAATGTCCACAATGCTGAAA	TGAAGTATCAGGGATACCAAGAAAG
Promoter	ACCGAGGAGGAGGAGGAGAA	GGGAGTGTCCAAACCTCTTT

## Abbreviations

M1 cells: murine myeloid progenitor M1 cells
Mml1: murine leukemia virus-induced myeloid leukemia 1
CTCF: CCCTC-binding factor
ChIP: chromatin immunoprecipitation
4C: circular chromosome conformation capture
DHS-seq: sequencing of DNase I hypersensitive sites
GAPDH: glyceraldehyde-3-phosphate dehydrogenase
4-OHT: 4-hydroxytamoxifen
