# Supplementary material for: Distal regulation of c-myb expression during IL-6-induced differentiation in murine myeloid progenitor M1 cells
Source: Cell Death Dis. 2016 Sep 8;7(9):e2364–. doi: 10.1038/cddis.2016.267 (PMC5059869; doi:10.1038/cddis.2016.267)
Supplement: Supplementary Information [file cddis2016267x1.doc]

**Supplementary Information**

**Figure S1 Expression of *c-myb* in M1 and differentiated M1 cells.**

Expression levels were determined by quantitative reverse transcription PCR. Total RNA samples were prepared from M1 cells, M1 cells treated with IL-6 for 12 or 24 h. Data are normalized to GAPDH expression. Error bars represent standard deviations (SD) (n=3). *** represents significant difference of expression compared to untreated M1 cells (P<0.001).

**Figure S2 Enrichment of H3K4me1 at the *c-myb* locus.**

ChIP using antibody specific for H3k4me1 was performed in M1 and differentiated M1 cells induced by IL-6. Primer sets specific for upstream regions of -25k, -28k and -56k were used for quantitative PCR of ChIP DNAs. Relative quantitation was carried out by the comparative threshold cycle (CT) method. Statistical analysis was performed using GraphPad Prism 5 software. Error bars represent standard deviations (SD) (n=3).

**Figure S3 Overexpression of Hoxa9 in M1 cells**

(A)Total cell extracts were prepared from untreated M1 cells and M1 cells transfected with control MIGR or MIGR-Hoxa9-ER vector in the presence of 200 or 500 nM 4-OHT, then immunoblot was performed to detect Hoxa9 expression. (B) Cytoplasmic and nuclear fractions were prepared from MIGR-Hoxa9-ER M1 cells and subjected to immunoblot to detect distribution of Hoxa9.

**Table S1. Potential interaction loci detected by 4C sequencing.**
