# Supplementary figures and images for: Distal regulation of c-myb expression during IL-6-induced differentiation in murine myeloid progenitor M1 cells
Source: Cell Death Dis. 2016 Sep 8;7(9):e2364–. doi: 10.1038/cddis.2016.267 (PMC5059869; doi:10.1038/cddis.2016.267)

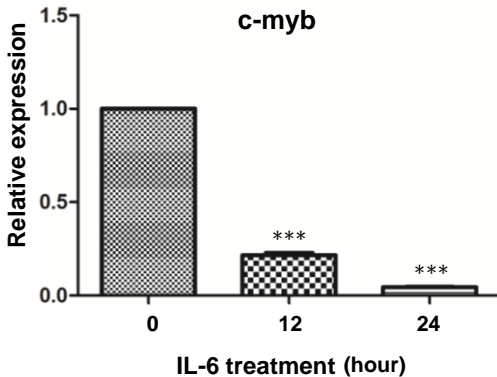

Supplement: Supplementary Figure S1 [file cddis2016267x2.pdf]

### H3K4me1

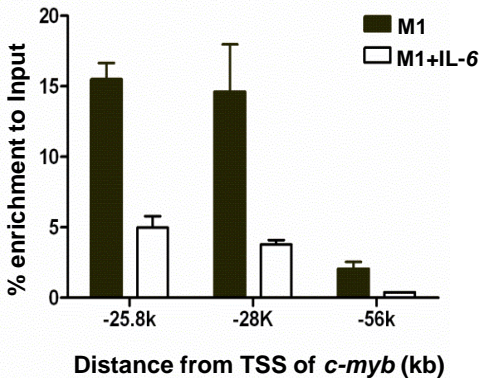

Supplement: Supplementary Figure S2 [file cddis2016267x3.pdf]

**A**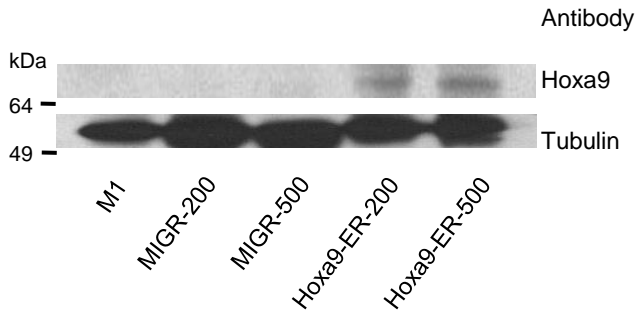**B**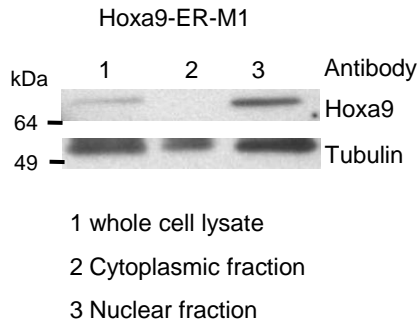

Supplement: Supplementary Figure S3 [file cddis2016267x4.pdf]
